# Deubiquitylating enzymes in neuronal health and disease

**DOI:** 10.1038/s41419-020-03361-5

**Published:** 2021-01-22

**Authors:** Fatima Amer-Sarsour, Alina Kordonsky, Yevgeny Berdichevsky, Gali Prag, Avraham Ashkenazi

**Affiliations:** 1 Department of Cell and Developmental Biology, Sackler Faculty of Medicine, Tel Aviv University, Tel Aviv, Israel; 2 School of Neurobiology, Biochemistry and Biophysics, the George S. Wise Faculty of Life Sciences, Tel Aviv University, Tel Aviv, Israel; 3 Sagol School of Neuroscience, Tel Aviv University, Tel Aviv, Israel

**Keywords:** Ubiquitylation, Neurological disorders

## Abstract

Ubiquitylation and deubiquitylation play a pivotal role in protein homeostasis (proteostasis). Proteostasis shapes the proteome landscape in the human brain and its impairment is linked to neurodevelopmental and neurodegenerative disorders. Here we discuss the emerging roles of deubiquitylating enzymes in neuronal function and survival. We provide an updated perspective on the genetics, physiology, structure, and function of deubiquitylases in neuronal health and disease.

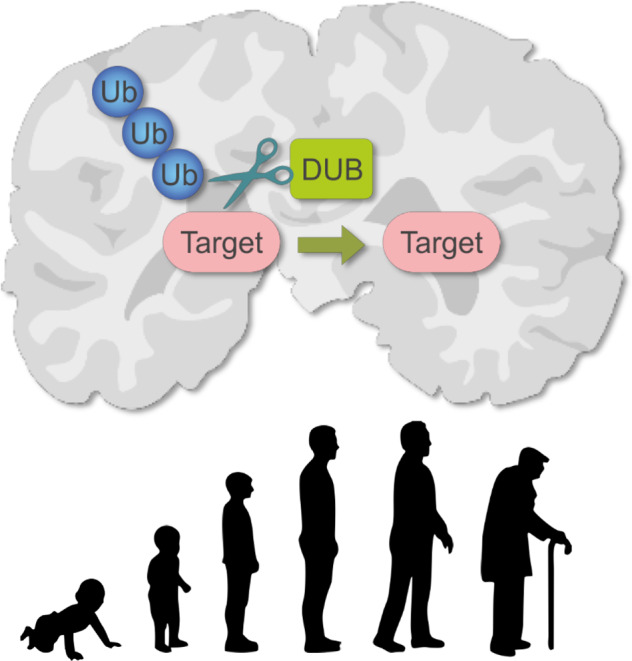

## Facts

- Deubiquitylation is essential for neuronal growth, function, and survival.
- Mutations in some DUBs are associated with neurodevelopmental and neurodegenerative disorders.
- DUBs influence learning and memory by regulating synaptic plasticity.
- DUBs affect neuronal vulnerabilities in stroke and neurodegenerative diseases.

## Open questions

- Do neuronal DUBs interact with different regulatory proteins or substrates compared to those in non-neuronal cells?
- How does the brain spatial expression pattern of DUBs correlate with specific neuronal functions?
- Do synaptic activities regulate DUB expression, function, and localization?
- Can we identify and employ pharmacological modulators of DUBs to treat neurological disorders?

## Introduction

**Fig. 1:**
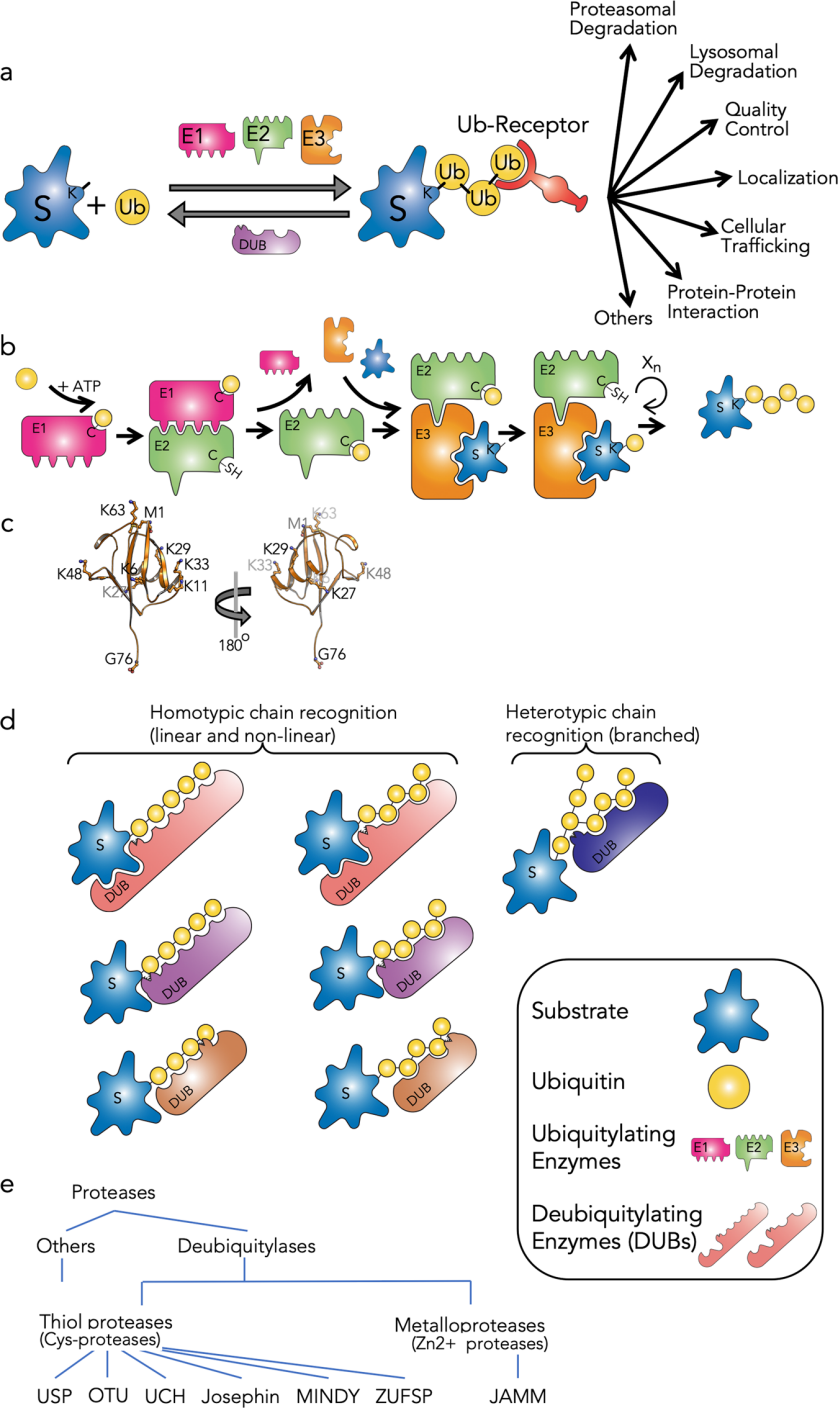
The ubiquitin system: ubiquitylation, ubiquitin recognition, and deubiquitylation. **a** A schematic representation of ubiquitylation/deubiquitylation events of target proteins and the cellular outcome. **b** The ubiquitylation cascade by three types of enzyme: ubiquitin-activating enzyme (E1), ubiquitin-conjugating enzyme (E2), and ubiquitin ligase (E3). **c** The seven lysine residues and the N-terminal methionine (M1) are projected on the structure of ubiquitin (PDB code 1UBQ^98^). **d** Principles of substrate recognition and ubiquitin cleavage by DUBs. **e**. Families of DUBs classified by sequence homology of their catalytic domains.

Ubiquitylation plays a pivotal role in cellular homeostasis by regulating proteasomal and lysosomal degradation, protein quality control, cellular trafficking and localization, protein–protein interactions, and many other cellular functions (Fig. 1a)^1–3^. The conjugation of ubiquitin to a target protein usually involves three types of enzyme: ubiquitin-activating enzyme (E1), ubiquitin-conjugating enzyme (E2), and ubiquitin ligase (E3)^1^. First, a catalytic cysteine of E1 forms a thioester bond with the C-terminus (G76) of ubiquitin, in an ATP-dependent reaction: namely, adenylation^4,5^. Ubiquitin is then transferred to a cysteine of E2^6–8^. Finally, E3 associates with specific target proteins and promotes the transfer of ubiquitin to a lysine (K) residue of the target (Fig. 1b)^1,3,6,9^. The monoubiquitylated target may undergo further ubiquitylation to form a multi-monoubiquitylated protein or the primed ubiquitin can be furthered ubiquitylated to form a chain known as a polyubiquitylated target. All seven lysine residues of ubiquitin (Lys6, Lys11, Lys27, Lys29, Lys33, Lys48, and Lys63) and its N-terminus (Met1) may serve as ubiquitylation sites, leading to the formation of homotypic or heterotypic (branched) ubiquitin chains (Fig. 1c, d)^1,3^. These different ubiquitylation forms are recognized by a large variety of ubiquitin receptors that contain ubiquitin-binding domains and decode the signals into different cellular responses in a spatio-temporal manner^10–14^ (Fig. 1a). Ubiquitylation is a highly dynamic process and is typically short-lived due to degradation or to the function of deubiquitylating enzymes (DUBs) that rapidly and efficiently reverse the signal^15–17^ (Fig. 1d). Interestingly, the transient characteristics of ubiquitin signals are also achieved by the association of E3s (or E2s) with DUBs^18,19^. In some cases, this association was even developed to a fusion of an E3 with a DUB to edit the ubiquitylation signal^20^.

In humans, approximately 90 DUBs are known, with diverse patterns of expression and abundance in different tissues^16^. Some DUBs recognize and cleave the peptide bond between the C-terminus of ubiquitin and the N-terminus of target proteins as a result of N-terminal ubiquitylation or linear ubiquitin chains^21^ (Fig. 1d), while other DUBs are restricted to the iso-peptide bonds as a result of the conjugation of ubiquitin to the side-chain residue of (usually) lysine at the targeted protein^15,16^ (Fig. 1d). A considerable number of DUBs are very large proteins that have acquired several domains during the course of evolution and display multifunctional activities^22,23^. DUBs recognize the C-terminus of ubiquitin and in many cases also specific residues in the vicinity of the conjugation site, which could be on the targeted protein or on ubiquitin itself in the case of cleavage within a polyubiquitin chain^15,16^. They can also possess several ubiquitin binding spots (or domains) that interact with the isoleucine 44 (I44) hydrophobic patch and/or other patches on ubiquitin in order to distinguish between different ubiquitin chains. DUBs are broadly classified as cysteine proteases or metalloproteases (Fig. 1e). The cysteine proteases include ubiquitin-specific proteases (USPs), ovarian tumor proteases (OTUs), ubiquitin C-terminal hydrolases (UCHs), Josephins, motif interacting with Ub-containing novel DUB family (MINDY), and Zn-finger and UFSP domain protein (ZUFSP). The zinc metalloproteases include JAB1/MPN/MOV34 (JAMMs).

The plethora of ubiquitin signals in the mammalian brain supports the idea that this type of post-translational modification is essential for neuronal function^24,25^. Because DUBs regulate ubiquitylation signals^16^, they play an important role in maintaining protein homeostasis (proteostasis) and signaling in neurons. Indeed, accumulating evidence in humans and in mice suggests that mutations in specific DUBs may lead to neurological abnormalities (Table 1). These studies promote our understanding of ubiquitin signaling in the development, function, and degeneration of the mammalian brain.

**Table 1:** Identified DUB mutations in humans with neurological disorders and proposed mechanisms in neuronal homeostasis.

DUB	Mutation in neurological disorder	Normal neuronal function	Reference
USP9X	Loss-of-function mutations in some forms of intellectual disability disorders, autism, and epilepsy.	Appears to be involved in synapse formation and neurogenesis.	^48,59^
OTUD7A	Gene deletion in some forms of intellectual disability disorders and seizures.	Supports synapse formation.	^55,56^
USP16	Triplicated gene in patients with Down’s syndrome.	Regulates cell-cycle progression and hematopoietic stem cells. Can affect neural progenitors.	^58^
USP7	Loss-of-function mutations in some forms of intellectual disability and autism spectrum disorders.	Regulates endosomal recycling. Seems to regulate the WASH complex and endosomal F-actin.	^60^
Ataxin-3	Expansion of the polyglutamine domain in patients with spinocerebellar ataxia type 3.	Regulates protein homeostasis (proteasome system and autophagy), and DNA damage response.	^72,74,75^
UCH-L1	Partial loss of the catalytic activity and other mutations in some patients with Parkinson’s, Alzheimer’s, and Huntington’s diseases.	Regulates free monomeric ubiquitin and synaptic function. Might be involved in synapse formation and neurotrophin receptor degradation (e.g., TrkB). Controls α-synuclein ubiquitylation state.	^87–89,91,95^

## Synaptic plasticity and neurodevelopment

**Fig. 2:**
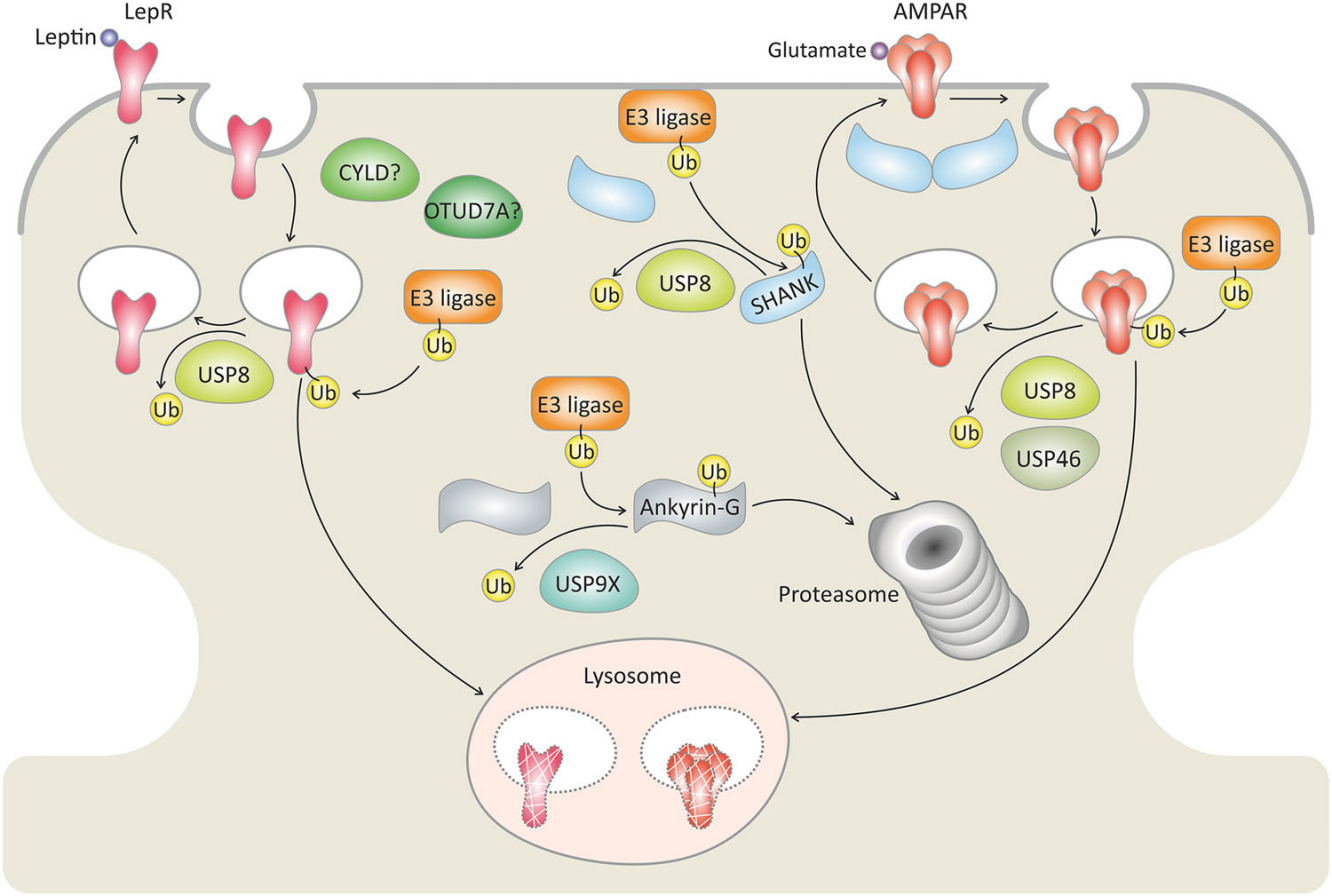
An emerging model for DUB regulation of synaptic plasticity. Activation of postsynaptic glutamate receptors controls neuron excitability. The postsynaptic density is enriched with a growing number of DUBs that antagonize E3-ligases and remove ubiquitin (Ub) chains, thereby protecting designated substrates from degradation by the lysosomes or the proteasome. Certain DUBs regulate the degradation and surface localization of the glutamate receptor, AMPAR. Moreover, a number of DUBs support the formation and function of the glutamatergic synapse by regulating the levels of scaffold proteins (SHANK and Ankyrin) and the leptin receptor (LepR). DUBs with yet unknown substrates are labeled with a question mark (?). The Ub symbol in this image indicates a polyubiquitin chain.

The excitatory synapse comprises a postsynaptic density and a presynaptic terminus. The latter is loaded with synaptic vesicles containing the neurotransmitter glutamate^26^. Glutamate released from the axonal presynaptic terminus binds to glutamate receptors at the postsynaptic density of the dendrite. This leads to ion influx, activation of voltage-gated ion channels, and activation of the specific signaling pathways leading to neuronal transmission^26^. DUBs are known to play diverse roles at the postsynaptic density. These include controlling the turnover of specific proteins involved in synaptic function, as well as ensuring monomeric ubiquitin availability (Fig. 2).

### DUB control of glutamate receptors

The postsynaptic density of the glutamatergic synapses is enriched with receptors and scaffolding proteins^26^. Members of one of the receptor families, the α-amino-3-hydroxy-5-methyl-4-isoxazolepropionic acid receptors (AMPARs), enable excitatory synaptic transmission in the brain. Synaptic plasticity, which is important for learning and memory, is influenced by the strength of synaptic transmission, which in turn correlates with the density of AMPARs at the synapse^26^. The amount of AMPARs on the cell surface is regulated by ubiquitylation and deubiquitylation. Both monoubiquitylation and polyubiquitylation are likely to exert an internalization signal^27^. However, the role of monoubiquitylation in lysosomal degradation of glutamate receptors is less clear in comparison to polyubiquitylation. For example, AMPARs undergo Lys63 polyubiquitylation by the HECT E3 ligase, NEDD4, resulting in AMPAR endocytosis and degradation in hippocampal neurons^28,29^. Conversely, the USP family member, USP46, is present in postsynaptic density fractions of hippocampal neurons and stabilizes cell-surface AMPAR levels by cleaving the Lys63 polyubiquitin chains^30^. USP46 knockout mice showed behavioral abnormalities, such as decreased exploratory behavior, which might be correlated with mental disorders^31^.

In the context of Alzheimer’s disease, it was recently shown that the aggregate-prone protein amyloid‐β induces NEDD4‐dependent AMPAR degradation, leading to synaptic weakening^32^. Moreover, amyloid‐β-exposed neurons showed an increased expression of NEDD4 and decreased levels of USP46^32^. This could suggest an antagonistic function of USP46 (in regard to NEDD4) in AMPAR recycling.

**Fig. 3:**
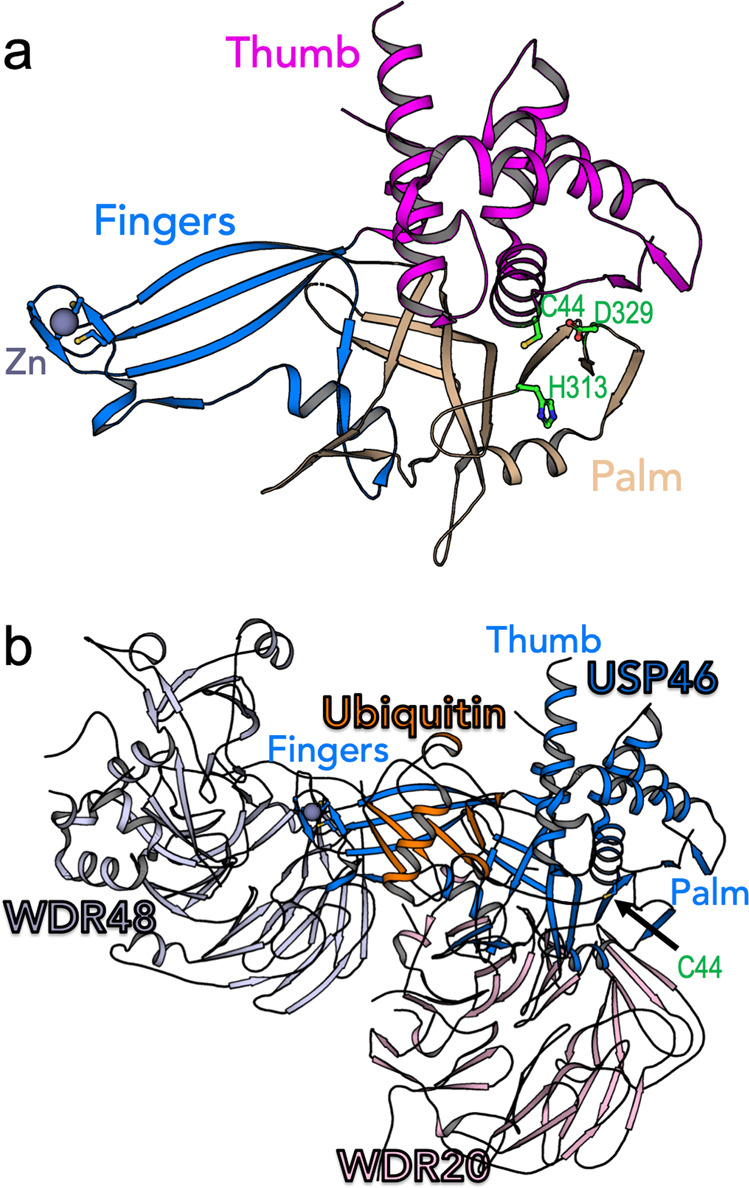
Structure of USP46 and its regulatory partners. **a** The structure of USP46 subdomains. Residues of the catalytic triad located at the interface between the palm and the thumb subdomains are shown as green sticks (PDB code 5L8H^40^). **b** A model of USP46 complex based on superposition of the structures of USP46 with covalent ubiquitin (Ub-VME) (PDB code 5L8H^40^) with the non-covalent complex of USP46:WDR20:WDR48 (PDB code 6JLQ^39^).

USP46, as well as other USPs, contains a region consisting of three subdomains: termed palm, fingers, and thumb^33^ (Fig. 3a). The active site that comprises the catalytic triad (residues Cys, Asp, and His) is located at the interface between the palm and thumb (Fig. 3a). The fingers subdomain binds and coordinates the cleaving ubiquitin in such a manner that its C-terminus becomes located in the active site^33^. In various USPs this binding induces conformational changes that realign the catalytic triad residues for catalysis^17^. The activity of USPs is regulated by several mechanisms, including the allosteric rearrangement of the catalytic triad^33^, post-translational modifications^34^, subcellular localization^35^, and/or by noncovalent interaction with other regulatory proteins such as WD40-repeat (WDR)^36^.

Interestingly, the *Caenorhabditis elegans* homologs of WDR20 and WDR48 (also known as UAF1) can bind to and activate USP46. The WDR protein effects on USP46 increase the stability of GLR-1, the *C. elegans* homolog of AMPAR^37^. We superimposed the crystal structures of USP46 in a non-covalent complex with WDR20 and WDR48 together with the covalent USP46–ubiquitin complex. This revealed that the two β-propellers bind remotely from the active site and do not interact with ubiquitin (Fig. 3b). It seems that, unlike the binding of other β-propeller proteins that realign the active site of DUBs, binding to the convex side of USP46 stimulates its activity via stabilization and a prolonged residence time of the ubiquitylated substrates^38^. Indeed, it has been demonstrated that the binding of WDR20 and WDR48 synergistically activates USP46^39^. Similarly, Sixma and colleagues demonstrated that WDR48 regulates USP12 by a two-step binding mechanism^40^.

Another USP member, USP8, is present in both somatic and dendritic compartments in hippocampal neurons and is also enriched in postsynaptic density fractions, suggesting that the protein might influence synaptic strength^41^. Upon N-methyl- d-aspartic acid receptor (NMDAR) activation, USP8 is dephosphorylated and facilitates the recycling of internalized AMPARs^41^. Recently, USP8 was also reported to be regulated by the hormone leptin that acts in the hippocampus^42^. Bland and colleagues suggested that leptin increases the expression of USP8, which in turn deubiquitylates the leptin receptor by cleaving Lys48-ubiquitin chains, among other (still unknown) chain types. This process is likely to be related to increased leptin receptor surface localization and synapse formation^42^.

### DUBs and scaffolding proteins at the postsynaptic density

SHANK proteins are scaffolding proteins of the postsynaptic glutamatergic synapses^43^ that bind to a variety of proteins at the postsynaptic density of excitatory synapses^44^. There are three main classes of SHANK proteins: namely, SHANK1, SHANK2, and SHANK3, and mutations in these proteins correlate with neurodevelopmental disorders. For example, mutations that alter SHANK3 protein levels were described in Phelan-McDermid syndrome, autism spectrum disorders, and schizophrenia^44^. A number of patients with high-functioning autism and schizophrenia show duplication of SHANK3^44^. USP8 stabilizes the levels of SHANK1 and SHANK3 proteins in hippocampal neurons, and thus increases the dendritic spine density^43^. USP8 also protects SHANK1 and SHANK3 from proteasomal degradation by cleaving the associated polyubiquitin chains^43^. Interestingly, SHANK3 was shown to regulate the striatal synaptic abundance of a USP member, CYLD, which specifically cleaves linear and Lys63-ubiquitin chains^45–47^. The substrates for deubiquitylation by CYLD at the postsynaptic density still remain unknown.

One of the approaches to identifying specific substrates for DUBs is a yeast two-hybrid screen. Some DUBs reveal a specific interaction with their substrates regardless of the ubiquitylation state (Fig. 1d) This increases the probability of finding these substrates in the yeast-two-hybrid system. Yoon and colleagues utilized this approach to identify the USP member USP9X as a DUB for the protein ankyrin-G in cortical neurons^48^. The function of ankyrin-G is to link plasma membrane proteins to the cytoskeleton^49^. Variants in the *ANK3* gene encoding ankyrin-G are associated with neurodevelopmental disorders, and ankyrin-G is enriched in postsynaptic density fractions and facilitates synapse formation^48^. USP9X cleaves polyubiquitin chains in ankyrin-G and protects ankyrin-G from ubiquitylation-dependent proteasomal degradation. Interestingly, USP9X phosphorylation enhances the DUB activity towards ankyrin-G and correlates with spine development^48^. This is important since loss-of-function mutations in the USP9X are correlated with intellectual disability, autism, and epilepsy^50^.

Another study by Paemka and colleagues demonstrated the nuclear receptors PRICKLE1/2 as targets for USP9X-mediated deubiquitylation^51^. Mutations in the genes coding for PRICKLEs are associated with seizures in humans and in mice, and the levels of PRICKLE2 were greatly reduced in mice lacking USP9X in the forebrain^51^. It has been demonstrated that PRICKLE is ubiquitylated by the ubiquitin E3 ligases HECT/NEDD4 family members SMURF1/2 for proteasome-mediated degradation^52^. Intriguingly, USP9X was also shown to interact with SMURF1 and to protect it from ligase activity-dependent self-degradation^53^. The association of DUBs with NEDD4 members is predicted to regulate their activity either by preventing their degradation or by reactivating their self-ubiquitylated basal inactive state^29^. However, USP9X directly interacts with PRICKLE1 and PRICKLE2 and cleaves their conjugated polyubiquitin chains^51^. The mechanism of PRICKLE’s regulation by USP9X thus seems to be highly complex and requires further mechanistic studies. Degrasyn (also known as WP1130) was serendipitously identified in a screen for JAK2 inhibitors. It pharmacologically inhibits the DUBs UCH37, USP5, USP14, and USP9X^54^. Degrasyn suppresses the seizure phenotype in PRICKLE mutant flies, suggesting that USP9X is involved in seizures and that modulating its activity might have potential as an anti-seizure modality^51^.

Microdeletions in 15q13.3 are rare conditions associated with intellectual disability and seizures^55,56^. One of the six genes commonly deleted in such neurological disorders is the OTU member OTUD7A, which cleaves Lys11-ubiquitin chains^55–57^. OTUD7A null mice possess a reduced number of functioning excitatory synapses and exhibit developmental delay. OTUD7A is localized to dendritic spines and is a positive regulator of dendritic spine density in cortical neurons^55,56^. It would be interesting to determine whether OTUD7A is selective for cortical synapses or also exerts its effects on hippocampal neurons.

Overall, it has become apparent that synaptic plasticity is regulated, at least in part, by a growing number of DUBs that form a network of key proteins responsible for regulating ubiquitin in the synapse. The contribution of DUBs in the postsynaptic density fraction to synaptic plasticity is depicted in Fig. 2.

### Other neurodevelopmental mechanisms mediated by DUBs

The formation of a functional excitatory synapse requires neuronal growth and maturation. These early stages in neuronal development are also regulated by certain DUBs (Table 1). The USP member USP16 regulates cell-cycle progression through deubiquitylation of histone H2A^58^. The self-renewal capacity of hematopoietic stem cells and neural progenitors is antagonized by USP16, which is triplicated in patients with Down’s syndrome^58^. Moreover, USP9X plays an important role in the self-renewal of hippocampal stem cells^59^. Interestingly, studies in USP9X knockout mice support the presence of an additional role for USP9X in the normal morphological development of the adult dentate gyrus^59^.

Other processes regulated by deubiquitylation, and linked to neurodevelopmental disorders, are those of cellular protein trafficking and endosomal recycling. In order to identify the probable DUBs involved, Hao and colleagues utilized tandem affinity purification of a specialized endosomal ubiquitin E3 ligase regulator, followed by liquid chromatography-tandem mass spectrometry (LC-MS/MS)^60^. This approach enabled them to identify the USP member USP7 as a regulator of WASH polyubiquitylation and endosomal F-actin levels in hypothalamic neurons^60^. WASH is an actin nucleation promoting factor essential for endosomal protein recycling^61^. In this context, loss-of-function mutations of USP7 were associated with intellectual disability and autism spectrum disorder that might result in part from defects in brain endosomal recycling^60^.

## Role of DUBs in neurodegeneration and stroke

**Fig. 4:**
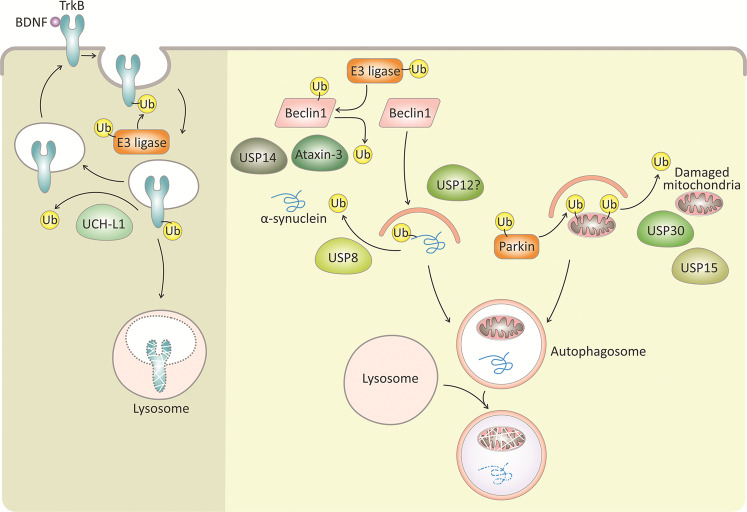
DUB involvement in neuron survival under normal conditions and in response to stressors. The cell survival tyrosine receptor kinase B (TrkB) is activated by BDNF and is ubiquitylated by E3-ligases for degradation. The DUB, UCH-L1, deubiqutylates TrkB and thereby regulates TrkB levels and surface localization. The accumulation of aggregate-prone proteins (for example, α-synuclein) and damaged mitochondria are associated with neuronal loss in neurodegenerative diseases, such as Parkinson’s disease. These proteins and damaged mitochondria are ubiquitylated by E3-ligases (for example, parkin ubiquitylation of damaged mitochondria) and are engulfed by autophagosomes for degradation in the lysosome. A number of DUBs antagonize autophagy-mediated degradation of damaged mitochondria (USP30 and USP15) and α-synuclein (USP8). Moreover, other DUBs (ataxin-3, USP14 and USP12) are involved in promoting neuronal autophagy. DUBs with yet unknown substrates are labeled with a question mark (?). The Ub symbol in this image indicates a polyubiquitin chain.

A number of neurodegenerative diseases, such as Alzheimer’s, Parkinson’s, and polyglutamine diseases, are associated with brain accumulation of toxic aggregate-prone proteins, which affect neuronal function and eventually lead to neuronal loss^62,63^. Another impaired brain condition, though of a very different kind, is ischemic stroke, which is characterized by disruption of the blood supply to the brain and vascular thrombus formation^63^. Such trauma to the brain causes neuronal death and is responsible for the subsequent neurological abnormalities, such as locomotor dysfunctions and impaired memory and learning skills^63^. Accumulating genetic and cellular evidence indicates a role for DUBs in neurodegeneration and stroke (Fig. 4).

### Brain pathologies and proteasome-resident DUBs

The proteasomal DUBs comprise three types of enzyme—USP14, UCHL5, and metalloprotease, PSMD14/RPN11^64^. These appear to be important for brain development. Depletion of the UCH family member, UCHL5, causes prenatal lethality in mice, with disorganized neuronal growth most evident in the areas of the forebrain, cerebellum, and midbrain^65^. Similarly, a genetic lesion in the gene encoding the USP member USP14 causes ataxia in mice, which is characterized by decreased brain development and reduced levels of free synaptic ubiquitin^66,67^. Moreover, USP14 was also shown to inhibit proteasome-mediated degradation by cleaving ubiquitin chains conjugated to substrates^68^. In this context, pharmacological inhibition of USP14 was neuroprotective, resulting in dose–response- increased proteasome activity and enhanced clearance of a number of toxic aggregate-prone proteins that have been implicated in neurodegenerative diseases^68,69^. Another example of neuroprotection was found in stroke, in which decreased expression of USP14 by microRNA or miR-124, as well as USP14 inactivation, was linked to neuron survival in the post-ischemic mouse brain^69^.

### DUBs and neuronal autophagy

Autophagy is a catabolic cell survival pathway that protects neurons from various stressors, such as aggregate-prone proteins and damaged mitochondria^70^. Upregulation of autophagy demonstrated beneficial effects in cell and animal models of polyglutamine, Alzheimer’s, and Parkinson’s diseases^70^. The process starts with the formation of autophagosomes, which engulf cytoplasmic proteins and organelles and direct them for degradation in the lysosome. One of the complexes involved in autophagosome initiation comprises beclin 1^71^. USP14 negatively regulates autophagy by cleaving the Lys63-ubiquitin chains and deubiquitylating beclin 1^71^. Remarkably, inhibiting USP14 resulted in an increase in both autophagy and proteasome activity, suggesting that USP14 could represent an attractive potential therapeutic target in neurodegenerative diseases^68,71^.

Ataxin-3, which is a member of the Josephine DUB family, has a preference for longer polyubiquitin chains and is known to interact with several ubiquitin ligases implicated in proteasome-mediated degradation^72^. However, it now appears that the functions of ataxin-3 extend beyond the proteasome system to other signaling pathways, including DNA damage and autophagy^72,73^. A polyglutamine expansion mutation in ataxin-3 causes spinocerebellar ataxia type 3 (SCA3), thereby providing a further link between ubiquitin-dependent protein quality control mechanisms and neurodegeneration (Table 1)^72,74^. The mutant ataxin-3 seems less effective than the wild type in deubiquitylating and therefore stabilizing beclin 1^75^. This may explain the reduced beclin 1 levels and compromised autophagy observed in the brains of SCA3 patients and related mouse models^74^. As a corollary, overexpression of beclin 1 can stimulate the autophagic flux and promote clearance of the mutant ataxin-3 in brains of SCA3 mouse models. This process was associated with a reduction in SCA3-related behavior and neuropathology^74^.

Another DUB that regulates autophagy in neurons is USP12. Aron and colleagues examined the mechanism of action of USP12 in primary neurons and stem cell-derived neuron models of Huntington’s disease^76^. They found that USP12 protects against mutant huntingtin toxicity, as evidenced by analyzing distinctive neuronal morphology and the disruption of plasma membrane as indications of cell death. Interestingly, the catalytic activity of USP12 appeared not to be required for the induction of autophagy flux and clearance of mutant huntingtin in these neurons^76^. These findings imply that a non-catalytic function of DUBs might be important for neuronal function. This could be of potential relevance when considering how to modulate the function of a DUB by targeting its catalytic site.

The USP family member USP8 is found in Lewy bodies, a neuropathological hallmark of Parkinson’s disease composed of lipids and α-synuclein inclusions^77,78^. Aggregate-prone α-synuclein is toxic to neurons and can be cleared via autophagy^70^. Autophagosomes engulf aggregate-prone proteins (e.g., mutant huntingtin, mutant ataxin-3, and α-synuclein) for degradation in the lysosome via autophagy receptor recognition of ubiquitin chain labeling, such as Lys63 chains^70^. USP8 interacts with α-synuclein and cleaves Lys63 ubiquitin chains in α-synuclein, thereby inhibiting α-synuclein degradation in the lysosome^78^.

In addition to their role in autophagy, there are a number of DUBs that facilitate or antagonize mitophagy, a specialized autophagy pathway that mediates the clearance of damaged mitochondria by lysosomes^79^. If left uncleared, defective mitochondria can represent a source of oxidative stress and DNA damage that has been related to Parkinson’s disease^79^. USP8 can facilitate mitophagy by regulating the E3-ubiquitin ligase, parkin^80^. The removal of Lys6- ubiquitin chains by USP8 stabilizes parkin levels in the cortical neurons, enhances parkin recruitment to damaged mitochondria, and ubiquitylates certain mitochondrial proteins (e.g., VDAC1 and TOMM20). USP8 appears to have opposing roles in the context of Parkinson’s disease by either stabilizing α-synuclein levels or promoting parkin-mediated mitophagy^78,80^.

Parkin-mediated mitophagy is antagonized by the USP family members USP30, USP15, and the short form of USP35 (Fig. 4)^81–83^. Since some of the studies exploring parkin-antagonizing DUBs were performed in non-neuronal cells with expression vectors, there has been an utmost need to develop neuronal relevant models to explore these DUBs. By utilizing induced neurons derived from embryonic stem cells, Harper and colleagues recently described the landscape of parkin-dependent ubiquitylation, and USP30-dependent deubiquitylation on mitochondria in these neurons^84^. USP30 is a mitochondrial resident enzyme, but is also found in the oxidative organelles, peroxisomes^81,85^. USP30 can process diverse polyubiquitin chain types and was found to be highly efficient in cleaving Lys6- ubiquitin chains^86^.

### The special case of UCH-L1

Ubiquitin C-terminal hydrolase L1 (UCH-L1), which belongs to the UCH DUB family, is enriched in the brain. UCH-L1 knockout mice exhibited neurodegeneration with aberrant synaptic structure and activity that could be attributed to reduced levels of free monomeric ubiquitin, since overexpression of ubiquitin in UCH-L1-deficient neurons restored the normal synaptic structure^87,88^. These results suggest that UCH-L1 plays an essential function in regulating ubiquitin homeostasis at the neuronal synapses^88^. In humans, UCH-L1 mutations are linked to neurodegenerative diseases, including spinocerebellar ataxia and Huntington’s disease, where the UCH-L1 polymorphism is linked to age at onset^89,90^. Moreover, UCH-L1 is downregulated in patients with Parkinson’s and Alzheimer’s diseases^91^. The functions of UCH-L1 are still enigmatic. It has been shown that UCH-L1 functions both as a DUB and as a ligase that extends Lys63-polyubiquitin chains on α-synuclein^92^. The ubiquitin ligase activity is enhanced by oligomerization of the enzyme^92^.

**Fig. 5:**
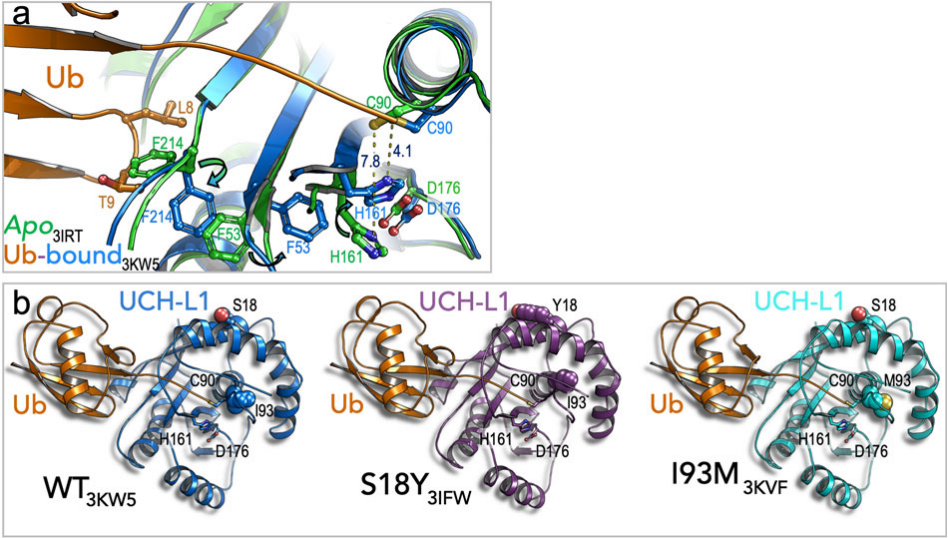
Structures of wild type and Parkinson’s disease-associated variants of UCH-L1. **a** Superposition of the structures of wild-type *apo* and covalent bound ubiquitin to UCH-L1 is shown (PDB code 3KW5^93^). Active site rearrangement and activation is due to ubiquitin binding. **b** Structures of the Parkinson’s disease variants, S18Y and I93M, are shown (PDB codes 3IFW and 3KVF) in comparison to the structure of wild-type UCH-L1 in complex with covalent ubiquitin.

Boudreaux and colleagues demonstrated that, like certain other DUBs, the active site of *apo* UCH-L1 is misaligned with the canonical catalytic triad (Fig. 5)^93^. They found that ubiquitin binding induces conformational changes that reorganize the enzyme active site into an active form. Superposition of the apo and the covalent ubiquitin bound structures demonstrated active site rearrangement as a consequence of the ubiquitin binding. Specifically, ubiquitin residues L8 and T9 push F214 of UCH-L1 towards F53, and the latter induces a new rotamer (other preferred structure) for H161 of the catalytic triad (Fig. 5a). This movement considerably reduces the distance between H161 and the catalytic C90 (from 7.8 to 4.1 Å). Interestingly, Boudreaux and colleagues also determined the structures of S18Y and I93M UCH-L1 mutants in covalent complex with ubiquitin at C90 (Fig. 5b)^93^. These mutants are associated with the risk to develop Parkinson’s disease in a contrasting manner. The I93M is a dominant mutation, which increases disease risk, while S18Y is a disease protective variant^92^. The structures show that the catalytic residues are aligned with a wild-type active triad (Fig. 5b). Nevertheless, isoleucine93 (I93) is located close to the active site and its substitution to methionine probably interferes with hydrolytic activity. However, serine18 (S18) is a surface residue located far from the active site (Fig. 5b) and therefore its substitution to tyrosine is not likely to interfere with hydrolytic activity per se. Strikingly, Liu and colleagues demonstrated that wild-type UCH-L1 forms dimers with a significant ligase activity; however, the S18Y variant decreases UCH-L1 dimerization, and consequently reduces ligase activity for α-synuclein^92^. They speculated that this phenomenon reduces susceptibility to Parkinson’s disease, and it is still unknown how UCH-L1-mediated ubiquitylation/deubiquitylation affects α-synuclein toxicity.

Our current knowledge of more substrates of UCH-L1 in neurons is limited. Brain-derived neurotrophic factor (BDNF) activation of tyrosine receptor kinase (Trk) regulates neuron survival and synaptic plasticity. Moreover, Trk neurotrophin receptors are regulated by ubiquitin modifications^94^. Recently, Guo and colleagues showed that UCH-L1 interacts with tyrosine receptor kinase B (TrkB)^95^. UCH-L1 mediates the deubiquitylation of TrkB by cleaving ubiquitin (probably polyubiquitin chains) and thereby inhibits TrkB degradation. These effects also correlate with the regulation of trafficking of surface TrkB in hippocampal neurons and BDNF-TrkB signaling^95^(Fig. 4).

## Future perspectives

It is generally accepted that alterations in the brain proteostasis network are linked to neurodegenerative and neurodevelopmental disorders. Recent progress on the underlying mechanisms suggests DUBs as regulators of proteostasis in neurons. Deubiquitylation appears to be a common signaling mechanism controlling synaptic plasticity, which is disrupted by DUB-associated neurodevelopmental mutations at the postsynaptic density. Since the accumulation of polyubiquitin chains triggers presynaptic assembly and growth^96^, it is plausible to assume that DUBs act on the presynaptic components. However, our knowledge regarding presynaptic ubiquitin regulation is still limited compared to that regarding postsynaptic density, and thus warrants further investigation.

Another open question relates to how the different DUBs coordinate with each other to ensure proper neuron function and response to stressors, such as aggregate-prone proteins and oxidative stress accumulation during aging. In particular, relating DUB mechanisms to spatial expression patterns in the mammalian brain might provide important information to answer this question. Such an analysis was recently performed for USP family members in granule neurons of the cerebellum^97^. These results may assist in targeting specific DUBs in brain pathologies involving differential neuronal vulnerabilities. In conclusion, we believe that future research into ubiquitin signaling holds great promise towards a better understanding of neuron homeostasis mechanisms, with the potential to design new therapeutic targets for various brain diseases.

## Supplementary information

Author contribution form
