# Supplementary material for: Deubiquitylating enzymes in neuronal health and disease
Source: Cell Death Dis. 2021 Jan 22;12(1):120. doi: 10.1038/s41419-020-03361-5 (PMC7822931; doi:10.1038/s41419-020-03361-5)
Supplement: Supplementary file 1 — Author contribution form [file 41419_2020_3361_MOESM1_ESM.pdf]

DECLARATION OF CONTRIBUTIONS TO ARTICLE

ADMC

Manuscript Number: CDDIS-20-4494R

Journal Name: *Cell Death & Disease* (the 'Journal')

Proposed Title of the Contribution: Deubiquitylating enzymes in neuronal health and disease (the 'Contribution)

Author(s): Fatima Amer-Sarsour, Alina Kordonsky, Yevgeny Berdichevsky, Gali Prag and Avraham Ashkenazi (the 'Authors')

For all *CDDis* articles, each person named as an author in the published version must be able to show he or she has contributed substantially to the article.

Authorship credit should be based on 1) substantial contributions to conception and design, acquisition of data, or analysis and interpretation of data; 2) drafting the article or revising it critically for important intellectual content; and 3) final approval of the version to be published. Authors should meet conditions 1, 2 and 3.

Any person who cannot be shown to have made a substantial contribution to the article cannot be listed as an author in the final version. The name of any person who is deemed to have made a minor contribution can, however, appear in the Acknowledgments section of the article.

Please complete the table below to indicate the contributions of all named authors to the manuscript.

Author Full Name:	Specification of Contribution to the Manuscript:
Fatima Amer-Sarsour	Wrote the paper
Alina Kordonsky	Wrote the paper
Yevgeny Berdichevsky	Wrote the paper
Gali Prag	Wrote the paper and developed the rational of the review.
Avraham Ashkenazi	Wrote the paper and developed the rational of the review.

Please complete the table below to indicate the contributions of all named authors to the figures.

Figure 1:

Designed the figure: Alina Kordonsky, Gali Prag and Avraham Ashkenazi

Figure 2:

Designed the figure: Avraham Ashkenazi and Fatima Amer-Sarsour

Figure 3:

Designed the figure: Gali Prag

Figure 4:

Designed the figure: Avraham Ashkenazi and Yevgeny Berdichevsky

Figure 5:

Designed the figure: Gali Prag

Figure 6:

Signed for and on behalf of the Author(s):	Print Name:	Date:
	Avraham Ashkenazi	04/12/2020
